# Web-Based Emotion Regulation Training for Sexual Health: Randomized Controlled Trial

**DOI:** 10.2196/50850

**Published:** 2024-04-03

**Authors:** Vinicius Jobim Fischer, Maila Rossato Holz, Joël Billieux, Gerhard Andersson, Claus Vögele

**Affiliations:** 1 Institute for Health and Behaviour Department of Behavioural and Cognitive Sciences University of Luxembourg Esch-sur-Alzette Luxembourg; 2 Conectare Neuropsi Porto Alegre Brazil; 3 Institute of Psychology University of Lausanne Lausanne Switzerland; 4 Department of Behavioural Science and Learning Department of Biomedical and Clinical Sciences Linköping University Linköping Sweden; 5 Department of Clinical Neuroscience Karolinska Institute Stockholm Sweden

**Keywords:** emotion regulation, internet, sexual health, FSFI, randomized controlled trial, intervention, psychosexual intervention, sexual disorder, sexual dysfunction, internet-based

## Abstract

**Background:**

Effective emotional regulation (ER) skills are important for sexual function, as they impact emotional awareness and expression during sexual activity, and therefore, satisfaction and distress. Emotion regulation interventions may offer a promising approach to improve sexual health. Web-based emotion regulation may be a therapeutic strategy for men and women with sexual health concerns. Nevertheless, there is a scarcity of intervention trials investigating its effects in this context, much less using the internet.

**Objective:**

This study aims to investigate the effects of a web-based emotion regulation training program for sexual function in both men and women.

**Methods:**

The participants were recruited based on their self-reported sexual problems, which for men was defined by a score of <25 on the International Index Erectile Function (IIEF) and for women by a score of <26.55 on the Female Sexual Function Index (FSFI). The final sample included 60 participants who were randomized to either a web-based emotion regulation training for sexual function or to a waitlist control group. The treatment consisted of an 8-week web-based emotion regulation training for sexual function. The participants were assessed at baseline, post intervention, and the 3-month follow-up.

**Results:**

Of the 60 participants included, only 6 completed all 3 assessment points (n=5, 20% in the treatment group and n=1, 5% in the waitlist control group) after receiving the intervention. At follow-up, there were no significant differences between groups in any measure. Among the intervention completers, large-to-moderate within-group effect sizes were observed between the assessment points on measures of emotion regulation, depression, lubrication, orgasm, thoughts of sexual failure, and abuse during sexual activity. The adherence rate was very low, limiting the generalizability of the findings.

**Conclusions:**

Participants who completed the intervention showed improvements in both sexual function domains and emotion regulation. Nonetheless, due to a high dropout rate, this trial failed to collect sufficient data to allow for any conclusions to be drawn on treatment effects.

**Trial Registration:**

ClinicalTrials.gov NCT04792177; https://clinicaltrials.gov/study/NCT04792177

## Introduction

### Background

Sexual dysfunctions involve difficulty in the ability to respond sexually or obtain sexual pleasure and are common and often disabling conditions [1]. Their etiology is multifactorial and encompasses biopsychosocial factors [2]. Throughout the lifespan, it has been estimated that 40% to 45% of adult women and 20% to 30% of adult men in the general population fulfill the criteria for at least 1 sexual dysfunction [3]. As an example, in the last available national survey in the United Kingdom, 51.2% of women and 41.6% of men who had sex in the last year reported at least 1 sexual problem [4].

The cognitive-affective model of sexual dysfunctions [5] posits that both healthy individuals and those with physical or mental conditions respond differently to sexual situations. While men and women without sexual dysfunctions respond to sexual situations with positive affect, expectations of success, and perception of control, those with sexual dysfunctions respond with anxiety, negative affect, and expectations of failure. In addition, preoccupations about erection and disengagement/thoughts about failure in men and lack of erotic thoughts in women have been found to be negatively correlated with sexual arousal. Across both sexes, sadness and disillusion are negatively associated with sexual arousal [6].

Emotion regulation (ER), the process by which emotions are generated, experienced, and used [7], has also been associated with sexual response cycle difficulties (arousal, lubrication, orgasm, pain, erection, and ejaculation) in both men and women [8-11]. Taking into consideration the high prevalence rates of emotion regulation difficulties and the high comorbidity of sexual disorders with other mental disorders [12], studies are needed to address ER interventions for sexual dysfunctions. The few studies investigating the effects of ER interventions have reported positive results, such as a reduction in sexual compulsivity, drug use, HIV risk behaviors, anxiety, and depression [13], and improvements in sexual functioning and quality of life [14].

Learning effective ER skills is important for sexual function, as they impact emotional awareness and expression during sexual activity, and, therefore, satisfaction and distress [15]. There is evidence that adaptive ER strategies are associated with better sexual function and mental health (anxiety and depression) [6] and reduced sensitivity and reactivity to negative stimuli [15].

Psychosexual interventions have been shown to be effective in treating sexual disorders [16]. They combine several interventions to address disorders experienced by both sexes. Generally, they are based on cognitive and behavioral interventions but can also include couple and systemic therapy components [17]. Most often, psychosexual therapies based on cognitive behavior therapy include (1) psychoeducation about sexual function, (2) cognitive challenges to negative sexual attitudes and distortions, and (3) behavioral activation via sex therapy–specific techniques [18]. The benefits of psychosexual therapies not only involve functional aspects but can also improve other domains of life, such as personal well-being and relationship quality and satisfaction [19,20].

Sexual dysfunctions are often perceived as sensitive, embarrassing, and potentially stigmatizing by those concerned. The relative anonymity of web-based approaches may offer an opportunity to attend therapy sessions in an environment that is perceived as safer and more private, while not requiring much investment of time for both the therapist and the client [21-23].

In general, web-based psychological interventions have been found to be effective in treating a range of conditions. The potential benefits of web-based interventions include their anonymity, availability, and convenience of pace/time [24]. In addition, web-based interventions can support active learning via interactive components [25].

To date, only a few studies have investigated the effects of web-delivered interventions for sexual dysfunctions. None of these studies included ER training. In one study, a treatment program for various sexual dysfunctions led to improvements in sexual functioning in 67% (n=39) of the participants, with improvements maintained at 1-month follow-up [26]. Similarly, treatment was found to be superior to a waitlist control condition in a study on internet-delivered sex therapy for erectile dysfunction [27] and in a study comparing web-delivered cognitive behavioral therapy (CBT) to an internet discussion forum control group [28].

Despite the importance of ER for sexual function, web-based ER interventions have, to our knowledge, not been developed and tested to any greater extent in people with sexual dysfunction. Therefore, this study aims to tackle this gap in the literature as the first ER web-based intervention designed to improve sexual function.

### Aim

This study aimed to investigate the effects of a web-based ER training program for sexual function in male and female participants experiencing sexual dysfunction named TREPS (Portuguese acronym for Emotion Regulation Training for Sexual Health). The training was provided in the form of an 8-week web-based program with therapist support via email on a secure treatment platform. We expected that TREPS group participants would show improvements in self-reported outcomes compared to the waitlist control group.

## Methods

### Overview

Participants were recruited from the general public via the internet. Advertisements concerning the project and invitations to take part in the study were disseminated using social media (Facebook and Instagram) for 4 months, targeting the Brazilian Portuguese–speaking population. Additionally, participants of a previous web-based survey known as the Sexual Health and Emotion Regulation (SHER) study who had volunteered to participate in future studies were also contacted.

The following inclusion criteria were used: (1) access to a computer/tablet or phone with an internet connection; (2) between 18 and 65 years of age; (3) fluent in Brazilian Portuguese; (4) self-reported sexual problems, as assessed in men by a score of <25 on the International Index Erectile Function (IIEF) and in women by a score of <26.5 on the Female Sexual Function Index (FSFI); and (5) in a stable relationship for at least the preceding last 3 months. The exclusion criteria were: (1) medical conditions that could interfere with the intervention, (eg, diabetes, cancer, and cardiovascular issues) or (2) currently receiving psychological or psychiatric treatment.

**Figure 1:**
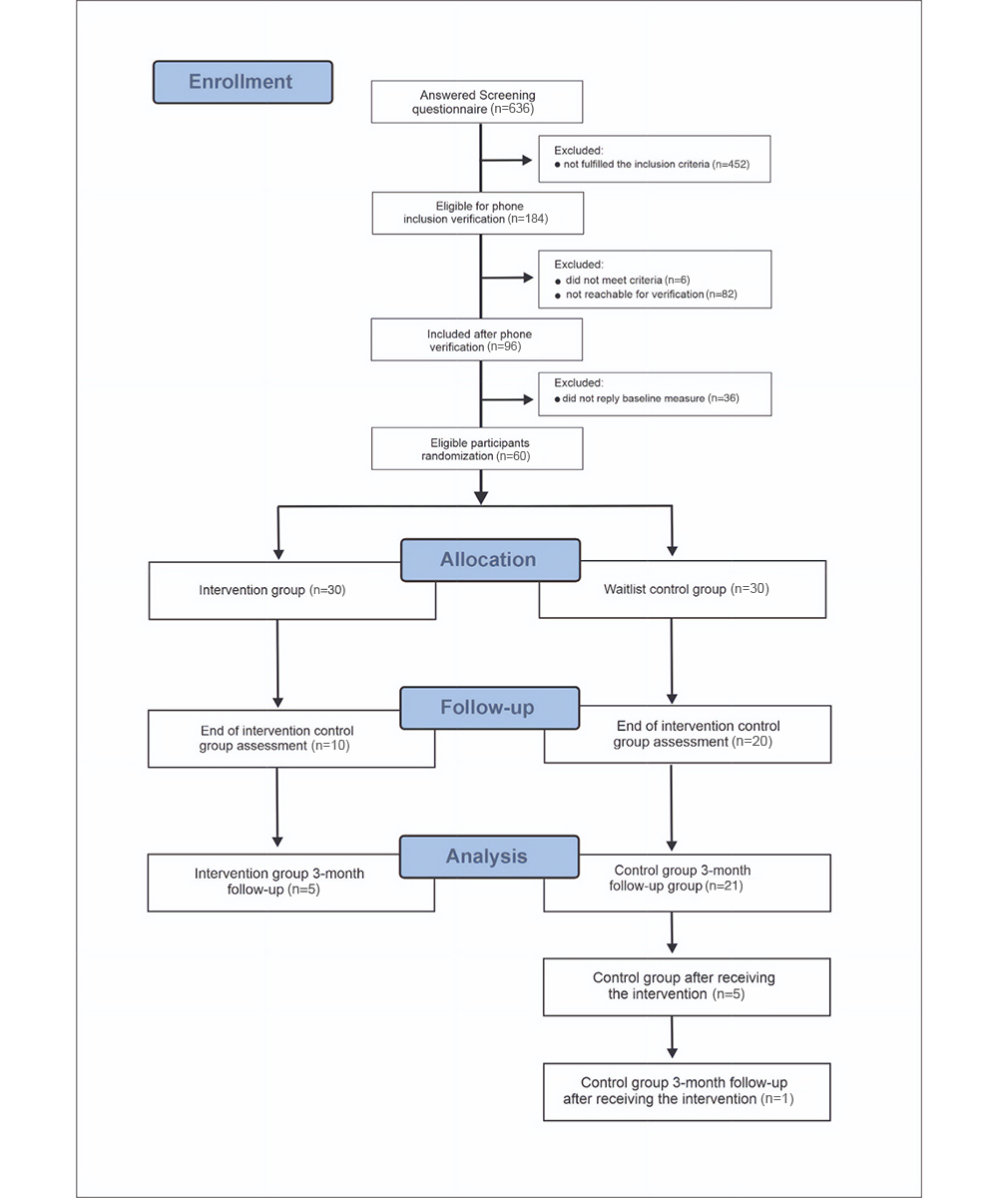
CONSORT (Consolidated Standards of Reporting Trials) flow diagram of the participant inclusion process.

Figure 1 illustrates the recruitment steps according to the CONSORT (Consolidated Standards of Reporting Trials) guidelines. After providing informed consent on the platform, the volunteers filled out a screening questionnaire to see if they met the inclusion criteria. For those whose responses indicated suitability for participation in the study, a phone contact was established to verify their eligibility and motivation to participate in the study. Following this, the included participants completed a baseline questionnaire.

Two separate block-randomization lists were created via a computer-generated (SealedEnvelope) block-randomization procedure (comprising a block size of 4 for 2 groups) with a 1:1 randomization ratio. The computer-generated sequence was generated by an independent researcher who was not involved in the trial. The actual allocation of participants to the TREPS intervention group or the waitlist control group was conducted by a clinical psychologist who was not involved in the project. Moreover, the researchers did not influence the allocation of the participants.

After randomization, the TREPS intervention group received the emotion regulation training for sexual function for 8 weeks, while the waitlist control group received no intervention. Both primary and secondary outcomes were assessed online on the study website at baseline, end of treatment, and the 3-month follow-up.

### Ethical Considerations

All study procedures were approved by the Ethics Review Panel of the University of Luxembourg (ERP-20-029 SHER). The study was also registered on ClinicalTrials.gov (NCT04792177), and the protocol was published [29]. Prior to taking part in this study, the participants provided electronic informed consent. Personal data collected in this study included information about sociodemographic characteristics and sexual and mental health. To ensure confidentiality, the data collected were pseudonymized and a unique identifier was generated so that participants’ identities could not be disclosed. No compensation was offered to the participants.

### Measures

#### Primary Outcome

The primary outcome measures were IIEF and FSFI scores for the male and female participants, respectively. The main reason for using these scores as main outcomes was that both have shown good reliability and validity for the Brazilian-speaking population [30,31]. For the IIEF, a cut-off score of 25 has been found to discriminate between diagnosis and no diagnosis [32], while for the FSFI, the respective score is 26.55 [33,34].

The IIEF is a 15-item, self-administered questionnaire for assessing sexual functioning in men [35]. Answers are given on a 6-point Likert scale. The IIEF encompasses 5 different domains of sexual functioning: erectile function, orgasm function, sexual desire, intercourse satisfaction, and overall satisfaction. Ferraz and Cicconelli [36] translated and adapted the scale to Brazilian Portuguese. Its psychometric properties have been reported by Gonzáles et al [30].

The FSFI is a 19-item questionnaire for the assessment of sexual functioning in women in the domains of sexual functioning (eg, sexual arousal, orgasm, satisfaction, and pain) [37]. Answers are provided using a 5-point Likert scale. Hentschel et al [31] translated and validated the FSFI into Portuguese.

#### Secondary Outcomes

The secondary outcome measures encompassed questionnaires about sexual function, mental health (anxiety and depression), ER, sexual self-perception, and thoughts during sexual activity. Sexual function was assessed with both the Sexual Quotient (SQ), using both the female [38] and male versions [39]. The SQ is a brief and comprehensive tool comprising 10 items that are answered on a scale from 0 (never) to 5 (always). It addresses general sexual function, stages of the sexual response cycle (desire, arousal, orgasm), and sexual satisfaction. Scores range between 0 and 100, with a score of ≤60 indicating sexual dysfunction.

Depression and anxiety symptoms were assessed via the Patient Heath Questionnaire-9 (PHQ-9) and the Generalized Anxiety Disorder-7 (GAD-7). Both instruments are frequently used self-report diagnostic tools for assessing mental health disorders.

The PHQ-9 [40] is a 9-item screening instrument that also provides an assessment of depression severity. The diagnostic validity of the tool has been established for its Brazilian version [41].

The GAD-7 [42] is a brief self-report measure specifically developed to assess generalized anxiety disorder. It has good reliability, as well as criterion, construct, factorial, and procedural validity. The Brazilian version has satisfactory psychometric properties [43].

The Difficulties in Emotion Regulation Scale (DERS) was used to assess ER. It covers several facets of ER, including difficulties relevant to an individual’s (1) acceptance of emotional responses, (2) ability to engage in goal-directed behavior under distress, (3) ability to control impulsive behaviors when distressed, (4) access to ER strategies, and (5) emotional clarity. Participants rate their degree of agreement with each statement on a scale from 1 (almost never; 0 to 10%) to 5 (almost always; 91 to 100%). The DERS was developed by Gratz and Roemer [44] and validated in Brazil by Miguel et al [45], with its psychometric properties confirmed by Cancian et al [46].

To assess sexual self-perception, the Sexual Self-Schema Scale (SSSS) was used [47]. It consists of 30 items assessing respondents’ perception of themselves as a sexual person compared to others of the same gender and age. Answers are provided using a 5-point Likert scale ranging from 1 (not at all descriptive of me) to 5 (very much descriptive of me). The psychometric properties of its Brazilian version have been found to be satisfactory [48].

To assess sexual thoughts during sexual activity, the Sexual Modes Questionnaire (SMQ) and its Automatic Thoughts subscale [49] were used. This self-report scale consists of 30 items in the male version and 33 items in the female version. Respondents are asked to rate the frequency, ranging from 1 (never) to 5 (always), with which they have experienced specific automatic thoughts during sexual activity. The psychometric properties of its Brazilian-adapted version have been evaluated [48].

### Intervention

The intervention consisted of 8 weekly modules delivered via the internet using a secure web-based contact handling system (eg, similar to internet banking) [50]. Each module consisted of a video presentation, texts, and an exercise (homework) to complete during the week. Access to the intervention modules was granted weekly on the same day, and participants were expected to report homework assignments to the therapist until the day before the next module. The participants were able to contact the therapist via the web-based platform and could expect a reply within 24 hours. No face-to-face or telephone contact with the research team was allowed during the intervention (except for technical problems). Table 1 summarizes the contents of the modules.

**Table 1:** Summary of the intervention modules.

Time frame	Module	Content
Week 1	Psychoeducation on sexual function	This module covers information about sexuality, the sexual response cycle (desire, excitement, plateau, orgasm, resolution), and the main difficulties that men and women may face in their sexual function, such as erectile dysfunction, premature ejaculation, desire disorders, pain disorders, and anorgasmia. It also differentiates the psychological characteristics of a functional sexual response from a dysfunctional sexual response and clarifies the difference between sexual function and sexual satisfaction [51].
Week 2	Psychoeducation on emotions and emotional regulation	This module defines emotions, their evolutionary aspects, emotion functions, emotion response cycles, emotion components (physical sensations, thoughts, and behaviors), and the long-term consequences of maintaining an emotional state for a longer period. It also defines pleasant and unpleasant emotions, the relationship between unpleasant emotions and avoidance behaviors, and avoidance strategies (emotional suppression, distraction, and behavioral avoidance) [52-54].
Week 3	Relaxation strategies: breathing and muscle relaxation	This module goes over the common physiological responses to anxiety and stress (eg, increases in heart rate, respiration, and muscle tension) and teaches 2 relaxation strategies: breathing relaxation and progressive muscle relaxation [52-54].
Week 4	Cognitive flexibility	This module refers to the rational component of emotion regulation. It aims to conceptualize and enhance cognitive flexibility. Moreover, the triad situation-thought-emotion is explained and detailed through the concepts of what distinguishes thoughts and interpretations, and what automatic thoughts and cognitive distortions are. The identification of negative thought patterns and the most common cognitive distortions related to sexuality are described [52-54].
Week 5	Nonjudgmental awareness	This module aims to teach participants to experience their emotions in the present moment in a nonjudgmental way. The module also differentiates between experiencing an unpleasant emotion from experiencing an unpleasant emotion resulting from negative beliefs and reactions (snowball effect) [52-54].
Week 6	Self-acceptance and compassion	This module focuses on 2 psychological concepts necessary for better emotion management: acceptance and self-compassion. These are important in order not to avoid emotional experiences and to diminish self-criticism associated with sexual difficulties [52-54].
Week 7	Emotion analysis	This module presents a step-by-step flowchart of how to identify emotions when experiencing them. By identifying emotions properly, we can facilitate effective emotion regulation. The flowchart comprises 6 items to pay attention to when identifying an emotion: (1) emotion, (2) event-trigger situation, (3) evaluation/interpretation, (4) physical sensations, (5) previous similar experiences, and (6) behavior [52-54].
Week 8	Sexual emotional exposures	This module summarizes all the previous modules and suggests a series of sexual experiences where participants pay attention to emotions experienced during the sexual activities (exposure). This gradual approach diminishes the risk of intense emotions and avoidant behaviors. By succeeding with the exposure and obtaining pleasure during the activities, feelings of danger/discomfort diminish, and more adaptive evaluations arise, facilitating the identification and modification of emotional behaviors.

The supporting clinician was a clinical psychologist and a European-certified psychosexologist. The waitlist control group received the same treatment at the end of the 3-month follow-up assessment.

### Data Analysis

The baseline data present normal univariate distribution according to skewness (−2 to 2) and kurtosis (−7 to 7). The sociodemographic characteristics of the 2 groups were compared using chi-square tests. Next, a 1-way analysis of variance (ANOVA) on outcome variables was performed to examine group differences at baseline. Mann-Whitney U tests were carried out to compare the 2 groups (intervention and control) at baseline, at the end of intervention (T1), and at follow-up (T2). Given the large amount of missing data (over 80%), we did not impute missing data or estimate missing data using mixed model analyses [33]. All participants who received the intervention and completed the 3 assessment points were compiled and analyzed for within-group efficacy estimation. A within-group nonparametric Fisher test was run to assess pre-post changes. Data were considered significant at a *P*<.05.

For the parametric analysis, the effect size was calculated using Cohen *d*. For the nonparametric analysis, effect sizes were calculated using η^2^ [55]. All statistical analyses were conducted with SPSS software (version 20.0; IBM Corp).

## Results

### Participants’ Characteristics

Initially, 60 participants completed the baseline questionnaires and were randomly assigned to one of 2 groups (intervention and waitlist control). Due to the high dropout rate and incomplete measures, we decided to merge the groups to compensate to some extent for the small sample size at post intervention. Table 2 shows the characteristics of the participants who fully adhered to the intervention and completed all the assessments (baseline, end of intervention, and 3-month follow-up).

**Table 2:** Participants’ sociodemographic characteristics.

Characteristics		Intervention group (n=30)		Control group (n=30)
Age (years), mean (SD)		30 (6.81)		31.3 (6.03)
Relationship duration (years), mean (SD)		7.60 (5.81)		6.66 (5.84)
**Country** **of** **residence, n (%)**				
	Brazil	28 (93)	28 (93)	
	Other	2 (7)	2 (7)	
**Gender, n (%)**				
	Female	29 (97)	29 (97)	
	Male	1 (3)	1 (3)	
**Sexual** **orientation, n (%)**				
	Heterosexual	22 (73)	26 (87)	
	Homosexual	0 (0)	1 (3)	
	Bisexual	7 (23)	3 (10)	
	Asexual	0 (0)	0 (0)	
	None of the above	1 (3)	0 (0)	
**Relationship status, n (%)**				
	Single	22 (73)	7 (23)	
	Informal partnership	7 (23)	3 (10)	
	Civic/formal partnership	0 (0)	6 (20)	
	Married	1 (3)	14 (47)	
**Education, n (%)**				
	Upper secondary	8 (27)	4 (13)	
	Tertiary/university	22 (73)	26 (87)	
**Occupation, n (%)**				
	Student (university)	4 (13)	0 (0)	
	Working full-time	16 (53)	14 (47)	
	Working part-time	4 (13)	4 (13)	
	Housewife/househusband	2 (7)	2 (7)	
	Owner/independent	3 (10)	7 (23)	
	Unemployed/jobseeker	1 (3)	2 (7)	

### Intervention Adherence

Intervention adherence was assessed by examining the number of participants who accessed the platform and opened the weekly modules. More participants completed the-end-of-intervention and follow-up assessments than those who completed all the intervention modules. Taking into consideration the total number of participants included in the study, adherence to the different modules varied between 14 (n=2) and 76.6% (n=23). The mean number of modules completed within the 8 weeks was 3.2 in the intervention group and 3 in the control group (after they had completed their intervention). Table 3 summarizes the number of participants who read and watched the modules.

**Table 3:** Adherence to the weekly modules.

Module	Group 1: intervention (n=30), n (%)	Group 2: control^a^ (n=20), n (%)
1	23 (77)	16 (80)
2	14 (47)	14 (70)
3	9 (30)	13 (65)
4	9 (30)	9 (45)
5	7 (23)	4 (20)
6	5 (17)	3 (15)
7	3 (10)	2 (10)
8	5 (17)	2 (10)

^a^When the intervention was offered to the second group, only 20 (67%) of the initial 30 participants completed the questionnaire and were therefore considered as the second intervention group.

An open-ended question section was used for the participants who completed the study, asking for suggestions and comments. A total of 3 topics emerged: (1) self-confidence, (2) influence on our emotional state of how we imagine others are seeing us, and (3) sexual repression during childhood and adolescence and its consequences in adult life. In addition, 1 participant suggested that the video materials should be longer.

### Between-Group Analysis

When comparing the intervention group (n=12, 40%) and the waitlist control group (n=16, 80%), statistically significant differences were found at baseline regarding the subscales about automatic thoughts of abuse (intervention: mean 21.47, SD 7.98; waitlist control: mean 15.40, SD 5.40; *P*=.004; *d*=0.81) and lack of partner attention (intervention: mean 13.20, SD 4.80; *P*=.03; *d*=0.61). At the end of the intervention (T1), differences were found regarding orgasm capacity and frequency in the intervention group (n=6, 20%) (mean 3.09, SD 2.54) and waitlist control (n=10, 50%) (mean 2.50, SD 1.47; *P*=.04; η^2^=2.58), as well as automatic thoughts of failure (intervention group: mean 4.43, SD 3.50; waitlist control: mean 9.33, SD 2.61; *P*=.01; η^2^=1.08).

Multimedia Appendix 1 presents the between-group comparisons at the 3 assessment time points.

### Within-Group Analyses

The intervention effect size calculation was conducted by combining all participants who completed the intervention and the 3-month follow-up assessment (n=5). The results indicated large within-group effect sizes for some of the outcome measures related to improvements in ER (DERS total score, η^2^=0.95; DERS goals, η^2^=0.94; DERS nonacceptance, η^2^=0.76), depression (PHQ-9, η^2^*=*0.73), orgasm (FSFI: orgasm η^2^*=*1.25), lubrification (FSFI: lubrication, η^2^*=*0.98), and failure automatic thoughts in a sexual context (EPA: failure, η^2^*=*0.95).

Medium effect sizes were found on all other factors of the DERS (η^2^*=*ranging from 0.50 to 0.68), anxiety (GAD-7, η^2^*=*0.43), arousal and partner connection in terms of the female version of the SQ (SQf; arousal and connection, η^2^*=*0.34), preliminaries (SQf: preliminaries, η^2^*=*0.38), abuse automatic thoughts in a sexual context (EPA: abuse, η^2^*=*0.38), and sexual self-schema (SSSS: loving/warm, η^2^*=*0.34)

Table 4 summarizes the within-group calculations.

Table 5 shows sexual function symptom scores for each participant who completed at least 2 assessment points.

**Table 4:** Within-group analysis after the follow-up.

Variables and scales		Mean (SD)	*P* value	Effect size (η^2^)
**FSFI^a^ sum**		15.93 (10.31)	N/A^b^	N/A
	FSFI: Desire	2.63 (1.48)	>.99	0
	FSFI: Arousal	2.56 (2.02)	N/A	N/A
	FSFI: Lubrication	2.68 (2.25)	.38	0.98
	FSFI: Orgasm	2.38 (1.91)	.14	1.25
	FSFI: Satisfaction	2.64 (1.71)	N/A	N/A
	FSFI: Pain	3.14 (2.54)	.91	0.12
**DERS^c^ sum**		99.65 (24.36)	.02	0.95
	DERS: Nonacceptance	15.73 (6.45)	.06	0.76
	DERS: Goals	16.92 (4.65)	.02	0.94
	DERS: Impulse	14.08 (5.39)	.21	0.51
	DERS: Awareness	15.65 (5.06)	.09	0.68
	DERS: Strategies	23.96 (8.12)	.21	0.50
	DERS: Clarity	13.31 (3.79)	.14	0.60
EPA^d^: Abuse		14.65 (7.10)	.37	0.38
EPA: Passivity control		7.65 (4.77)	.57	0.23
EPA: Negative self-image		13.54 (6.06)	.85	0.08
EPA: Failure		7.46 (3.41)	.03	0.95
EPA: Lack of partner attention		10.69 (5.15)	.95	0.03
EPA: Erotic thoughts		12.85 (5.39)	.80	0.10
SSSS^e^: Direct/outspoken		0 (0)	.85	0.09
SSSS: Loving/warm		0 (0)	.41	0.34
SSSS: Reserved/conservative		0 (0)	.80	0.10
**SQf^f^ sum**		54.31 (26.73)	>.99	0.01
	SQf: Sexual desire and interest	7.31 (4.36)	.66	0.19
	SQf: Preliminaries	3.27 (1.82)	.37	0.38
	SQf: Arousal and partner connection	5.81 (3.08)	.41	0.34
	SQf: Comfort	6.19 (3.24)	.75	0.14
	SQf: Orgasm and satisfaction	4.58 (2.98)	.81	0.11
**PHQ-9^g^ sum**		13.65 (7.94)	.08	0.73
	PHQ-9: Suicide	N/A	.53	0.27
	PHQ-9: Q10	N/A	.45	0.32
GAD-7^h^: Sum		10.27 (6.13)	.34	0.43

^a^FSFI: Female Sexual Function Index.

^b^N/A: not applicable.

^c^DERS: Difficulties in Emotion Regulation Scale.

^d^EPA: Automatic Thoughts subscale, Sexual Modes Questionnaire.

^e^SSSS: Sexual Self-Schema Scale.

^f^SQf: Sexual Quotient: female version.

^g^PHQ-9: Patient Heath Questionnaire-9.

^h^GAD-7: Generalized Anxiety Disorder-7.

**Table 5:** Sexual function scores of participants who completed at least 2 assessments.

Participant	Baseline (FSFI^a^/SQ^b^)	End of intervention (FSFI/SQ)	3-month follow-up (FSFI/SQ)
1	3.2/86	30.3/80	N/A^c^
2	17.6/40	N/A	2/28
3	11/36	16.3/50	N/A
4	12.3/32	N/A	15.9/N/A
5	20.9/48	19.7/46	N/A
6	22.1/58	26.3/62	30.5/70
7	11.4/32	26.5/56	22.7/56
8	2.4/80	3.2/84	N/A
9	21.4/60	30.5/64	N/A
10	20.8/40	31.4/72	18.2/46
11	22.3/90	22.7/86	N/A

^a^FSFI: Female Sexual Function Index.

^b^SQ: Sexual Quotient.

^c^N/A: not applicable.

## Discussion

### Principal Findings

This study aimed to investigate the effects of a web-based ER training program for sexual function. The trial faced a substantial dropout rate, but the preliminary results suggest that the TREPS protocol provided some improvements in relation to sexual function, albeit mainly for mental health and ER abilities. Although no significant effects were seen in the main outcome measures after the 3-month follow-up in the controlled between-group analyses, large and moderate within-group effect sizes were found for a range of components in the within-group analyses.

First, based on complete case analyses and the combined sample, large effects were found for the ER assessment as well as the depression and anxiety measures. Internet-based ER interventions for depression and emotional disorders have been shown to be efficacious with moderate-to-large between-group effect sizes [56-58]. The improvements we observed in this study are in line with those reported for face-to-face interventions focusing on mood disorders [59].

Regarding sexuality, moderate-to-large effects were found regarding the sexual components of arousal/lubrication and orgasm but not in other domains or the overall sexual function score. These sexual domains are more susceptible to individual sex practices and may have benefitted most from the intervention. This is plausible, as frustration with partners was frequently reported in participants’ responses to homework activities, and such components are associated with sexual desire, arousal, and satisfaction [60,61]. Regarding automatic thoughts in sexual activity, there were large intervention effects concerning failure and disengagement thoughts and a moderate effect concerning abuse thoughts. These findings are particularly relevant since both aspects have been suggested as the main cognitive components of sexual dysfunction in women [62,63].

Against our expectations, several factors did not allow us to strictly adhere to the published study protocol [29]. The 2 main changes concerned the follow-up, which was reduced from 6 to 3 months, and the conversion of the initially planned waitlist group into a second intervention group, assessed after receiving the intervention and 3 months later. These changes were implemented to counteract the high dropout rate in the intervention group.

Even though high attrition rates in web-based interventions for sexual function have been described in the past [29,63], the dropout rate in this study superseded those previously reported. When designing the study, precautions regarding adherence were made as suggested in the literature, such as a weekly scheduled treatment program, reminders, direct feedback, and positive messaging upon assignment completion [64,65].

Since no feedback was obtained from nonadherers, any conclusions must remain speculative. Our main hypothesis concerns the COVID-19 pandemic. The intervention was carried out during the COVID-19 outbreak, which affected Brazilians to a larger extent than other countries—by October 2021, Brazil had over 21.6 million infections and over 600 thousand deaths [66]. The number of completed modules was low, which is likely to have impacted the overall response to treatment. Among the participants, only 14% (n=7) accessed the last module, and less than 22% (n=11) accessed more than half of the intervention. Other web-based intervention studies focusing on male sexual disorders (erectile dysfunction) have reported similar findings. For instance, in one study, only 8% of the participants reached the final module, with 54% accessing just up to module 4 (out of 7) [28]. In a similar vein, a 70% dropout rate was reported for another web-based CBT program on sexual dysfunction [19].

Given the limited availability of internet interventions addressing sexual problems, further research is needed to understand the reasons behind the observed high dropout rates in the existing literature. As speculations, potential explanations can be made related to the nature of the sexual dysfunction, expectations, or outcomes.

Concerning the nature of the problem, given the personal and often taboo nature of sexuality and intimacy, it is reasonable to consider that worries about privacy or social stigma may significantly influence participant adherence to these interventions. Regarding treatment expectations, the concerned population often does not identify sexual problems as biopsychosocial complex problems. This perspective can lead to unrealistic expectations regarding the duration and effectiveness of interventions.

Regarding outcomes, individuals with sexual problems usually have misconceptions about sexual function and response. It is possible that if sufficient improvement is perceived after the psychoeducational phase (usually initial modules) of internet interventions, they might decide not to continue with the intervention.

Although adherence was low in this trial, the treatment remains easily scalable. The web-based intervention can be easily disseminated or updated at almost no cost and, therefore, is much cheaper than traditional face-to-face specialized treatments. Special attention must be paid to improving adherence.

Another important aspect concerns the unequal participation of men and women. While the TREPS training was developed for both men and women, only women participated in the study. This may be attributed to a higher level of interest among women in research studies [59,67] and related to how we recruited the participants. In addition to social media advertisements and contact with participants from a previous questionnaire study who volunteered to participate in future studies, we credited the female sample to a Brazilian female sexual health social media influencer (mainly followed by women), who shared the study on her social media account.

### Limitations

First, the high dropout rate precludes any significant conclusions to be drawn from the results. Significant attrition should be addressed as a risk factor in future similar web-based ER training for sexual function. In addition, and in contrast to the potential advantages of web-based interventions (eg, anonymity, ease of access, etc), the high dropout rates in programs of this kind may indicate a serious limitation to the suitability of web-based training concerning sexual health. Second, most participants were highly educated, which affects the generalizability of the results and their scalability. Third, since the inclusion criteria were based on self-report measures, response bias and possible failures to detect interfering medical conditions not known by the participants may have occurred. Fourth, we did not assess participants’ expectations regarding the effects of the training, so it remains unclear as to whether these impacted the results.

### Recommendations

For future studies, we recommend some changes in the study design and treatment components. Regarding recruitment, it would be advisable to have a referral system from a specialized sexual or mental health care service. Perhaps the recommendation of the training from a health service could improve adherence. Regarding the intervention, attention should be paid to the points raised by the intervention adherers, such as increasing the length and the amount of information provided per module. Moreover, there are currently no comparisons between web-based ER training for sexual health and face-to-face interventions. For this reason, comparisons between different intervention delivery methods such as face-to-face individual or group format interventions and web-based programs will be important. Future trials would also benefit from the inclusion of partner-related assessments.

### Conclusions

Our preliminary results suggest that web-based ER training may be an effective supplementary or alternative treatment for sexual disorders. Completers of the intervention presented improvements both in sexual function domains as well as in emotion regulation. Nonetheless, due to a high dropout rate, this trial failed to collect sufficient data to allow for any conclusions to be drawn on treatment effects.

Despite its limitations, this study presents valuable information about web-based ER interventions for both mental and sexual health treatments. The improvements in ER skills, mental health, and specific sexual domains call for the further development and evaluation of such interventions. Further research is needed to better understand the feasibility and scalability of similar protocols and how male participants would respond to them.

## Appendix

Multimedia Appendix 1 Between-group comparisons at baseline, end of intervention, and 3-month follow-up.

Multimedia Appendix 2 CONSORT eHEALTH Checklist (V 1.6.2).

## Abbreviations

ANOVA: analysis of variance
CBT: cognitive behavioral therapy
CONSORT: Consolidated Standards of Reporting Trials
DERS: Difficulties in Emotion Regulation Scale
ER: emotion regulation
FSFI: Female Sexual Function Index
GAD-7: Generalized Anxiety Disorder-7
IIEF: International Index Erectile Function
PHQ-9: Patient Heath Questionnaire-9
SHER: Sexual Health and Emotion Regulation
SMQ: Sexual Modes Questionnaire
SQ: Sexual Quotient
SQf: Sexual Quotient: female version
SSSS: Sexual Self-Schema Scale
TREPS: Emotion Regulation Training for Sexual Health (translated from Portuguese)

## Data Availability

The study data set generated during this investigation is not publicly available due to ethical concerns. Both the data set and the study metadata are available from the corresponding author upon request.

## References

[1] American Psychiatric Association (2013). Diagnostic and Statistical Manual of Mental Disorders (DSM-5).

[2] Willi J, Burri A (2015). Emotional intelligence and sexual functioning in a sample of Swiss men and women. J Sex Med.

[3] Lewis RW, Fugl-Meyer KS, Corona G, Hayes RD, Laumann EO, Moreira ED, Rellini AH, Segraves T (2010). Definitions/epidemiology/risk factors for sexual dysfunction. J Sex Med.

[4] Mitchell KR, Mercer CH, Ploubidis GB, Jones KG, Datta J, Field N, Copas AJ, Tanton C, Erens B, Sonnenberg P, Clifton S, Macdowall W, Phelps A, Johnson AM, Wellings K (2013). Sexual function in Britain: findings from the third National Survey of Sexual Attitudes and Lifestyles (Natsal-3). Lancet.

[5] Barlow DH (1986). Causes of sexual dysfunction: The role of anxiety and cognitive interference. J Consult Clin Psychol.

[6] Fischer VJ, Andersson G, Billieux J, Infanti A, Vögele C (2023). The role of emotion regulation strategies for sexual function and mental health: a cluster analytical approach. J Sex Marital Ther.

[7] Lumley MA, Cohen JL, Borszcz GS, Cano A, Radcliffe AM, Porter LS, Schubiner H, Keefe FJ (2011). Pain and emotion: a biopsychosocial review of recent research. J Clin Psychol.

[8] Madioni F, Mammana LA (2001). Toronto Alexithymia Scale in outpatients with sexual disorders. Psychopathology.

[9] Michetti PM, Rossi R, Bonanno D, De Dominicis C, Iori F, Simonelli C (2007). Dysregulation of emotions and premature ejaculation (PE): alexithymia in 100 outpatients. J Sex Med.

[10] Berenguer C, Rebôlo C, Costa RM (2019). Interoceptive awareness, alexithymia, and sexual function. J Sex Marital Ther.

[11] Dubé JP, Corsini-Munt S, Muise A, Rosen NO (2019). Emotion regulation in couples affected by female sexual interest/arousal disorder. Arch Sex Behav.

[12] Jonusiene G, Griffioen T (2013). Psychiatric disorders and sexual dysfunctions. The EFS and ESSM Syllabus of Clinical Sexology.

[13] Parsons JT, Rendina HJ, Moody RL, Gurung S, Starks TJ, Pachankis JE (2017). Feasibility of an emotion regulation intervention to improve mental health and reduce HIV transmission risk behaviors for HIV-positive gay and bisexual men with sexual compulsivity. AIDS Behav.

[14] de Ornelas Maia ACC, Sanford J, Boettcher H, Nardi AE, Barlow D (2017). Improvement in quality of life and sexual functioning in a comorbid sample after the unified protocol transdiagnostic group treatment. J Psychiatr Res.

[15] Rosen NO, Bergeron S (2019). Genito-pelvic pain through a dyadic lens: moving toward an interpersonal emotion regulation model of women's sexual dysfunction. J Sex Res.

[16] Frühauf S, Gerger H, Schmidt HM, Munder T, Barth J (2013). Efficacy of psychological interventions for sexual dysfunction: a systematic review and meta-analysis. Arch Sex Behav.

[17] Kingsberg SA, Althof S, Simon JA, Bradford A, Bitzer J, Carvalho J, Flynn KE, Nappi RE, Reese JB, Rezaee RL, Schover L, Shifrin JL (2017). Female sexual dysfunction-medical and psychological treatments, committee 14. J Sex Med.

[18] Meana M, Jones S (2011). Developments and trends in sex therapy. Adv Psychosom Med.

[19] McCabe MP, Price E, Piterman L, Lording D (2008). Evaluation of an internet-based psychological intervention for the treatment of erectile dysfunction. Int J Impot Res.

[20] McCabe M, Price E (2008). Internet-based psychological and oral medical treatment compared to psychological treatment alone for ED. J Sex Med.

[21] Cooper A, Scherer C, Mathy RM (2001). Overcoming methodological concerns in the investigation of online sexual activities. Cyberpsychol Behav.

[22] Taylor CB, Luce KH (2016). Computer- and internet-based psychotherapy interventions. Curr Dir Psychol Sci.

[23] Hummel SB, van Lankveld JJ, Oldenburg HS, Hahn DE, Broomans E, Aaronson NK (2015). Internet-based cognitive behavioral therapy for sexual dysfunctions in women treated for breast cancer: design of a multicenter, randomized controlled trial. BMC Cancer.

[24] Andersson G, Titov N, Dear BF, Rozental A, Carlbring P (2019). Internet-delivered psychological treatments: from innovation to implementation. World Psychiatry.

[25] Andersson G, Berger T (2021). Internet approaches to psychotherapy: mpirical findings and future directions. Handbook of Psychotherapy and Behavior Change.

[26] Van Diest SL, Van Lankveld JJDM, Leusink PM, Slob AK, Gijs L (2007). Sex therapy through the internet for men with sexual dysfunctions: a pilot study. J Sex Marital Ther.

[27] van Lankveld JDM, Leusink P, van Diest S, Gijs L, Slob AK (2009). Internet-based brief sex therapy for heterosexual men with sexual dysfunctions: a randomized controlled pilot trial. J Sex Med.

[28] Andersson E, Walén C, Hallberg J, Paxling B, Dahlin M, Almlöv J, Källström R, Wijma K, Carlbring P, Andersson G (2011). A randomized controlled trial of guided Internet-delivered cognitive behavioral therapy for erectile dysfunction. J Sex Med.

[29] Fischer VJ, Andersson G, Billieux J, Vögele C (2021). A randomized controlled trial of an Internet-based emotion regulation intervention for sexual health: study protocol. Trials.

[30] Gonzáles AI, Sties S, Wittkopf P, Mara LS, Ulbrich AZ, Cardoso FL, Carvalho T (2013). Validation of the International Index of Erectile Function (IIFE) for use in Brazil. Arq Bras Cardiol.

[31] Hentschel H, Alberton DL, Capp E, Goldim JR, Passos Ep (2007). Validação do Female Sexual Function Index (FSFI) para uso em língua portuguesa. Rev HCPA. Rev HCPA.

[32] Rosen RC, Cappelleri JC, Gendrano N (2002). The International Index of Erectile Function (IIEF): a state-of-the-science review. Int J Impot Res.

[33] Jakobsen JC, Gluud C, Wetterslev J, Winkel P (2017). When and how should multiple imputation be used for handling missing data in randomised clinical trials - a practical guide with flowcharts. BMC Med Res Methodol.

[34] Wiegel M, Meston C, Rosen R (2005). The female sexual function index (FSFI): cross-validation and development of clinical cutoff scores. J Sex Marital Ther.

[35] Rosen RC, Riley A, Wagner G, Osterloh IH, Kirkpatrick J, Mishra A (1997). The international index of erectile function (IIEF): a multidimensional scale for assessment of erectile dysfunction. Urology.

[36] Ferraz M, Ciconelli M (1998). Tradução e adaptação cultural do índice internacional de função erétil para a língua portuguesa. Rev Bras Med.

[37] Rosen R, Brown C, Heiman J, Leiblum S, Meston C, Shabsigh R, Ferguson D, D'Agostino R (2000). The Female Sexual Function Index (FSFI): a multidimensional self-report instrument for the assessment of female sexual function. J Sex Marital Ther.

[38] Abdo C (2006). Elaboração e validação do quociente sexual, versão feminina: uma escala para avaliar a função sexual da mulher. Rev Bras Med.

[39] Abdo CH (2007). The male sexual quotient: a brief, self-administered questionnaire to assess male sexual satisfaction. J Sex Med.

[40] Spitzer RL, Kroenke K, Williams J B (1999). Validation and utility of a self-report version of PRIME-MD: the PHQ primary care study. Primary Care Evaluation of Mental Disorders. Patient Health Questionnaire. JAMA.

[41] de LOF, Vilela MA, Crippa JA, Loureiro SR (2009). Study of the discriminative validity of the PHQ-9 and PHQ-2 in a sample of Brazilian women in the context of primary health care. Perspect Psychiatr Care.

[42] Spitzer RL, Kroenke K, Williams JBW, Löwe B (2006). A brief measure for assessing generalized anxiety disorder: the GAD-7. Arch Intern Med.

[43] Moreno AL, DeSousa DA, Souza AMFLP, Manfro GG, Salum GA, Koller SH, Osório FL, Crippa JAS (2016). Factor structure, reliability, and item parameters of the brazilian-Portuguese version of the GAD-7 questionnaire. Temas Psicol.

[44] Gratz KL, Roemer L (2004). Multidimensional assessment of emotion regulation and dysregulation: development, factor structure, and initial validation of the Difficulties in Emotion Regulation Scale. J Psychopathol Behav Assess.

[45] Miguel FK, Giromini L, Colombarolli MS, Zuanazzi AC, Zennaro A (2017). A Brazilian investigation of the 36- and 16-item difficulties in emotion regulation scales. J Clin Psychol.

[46] Cancian ACM, Souza LASD, Silva VHPE, Machado WDL, Oliveira MDS (2019). Psychometric properties of the Brazilian version of the Difficulties in Emotion Regulation Scale (DERS). Trends Psychiatry Psychother.

[47] Hill DB (2007). Differences and similarities in men's and women's sexual self-schemas. J Sex Res.

[48] Lucena B (2019). Fatores cognitivos na função sexual: adaptação transcultural e estudo psicométrico de instrumentos de medida em sexualidade [Doctoral thesis].

[49] Nobre PJ, Pinto-Gouveia J (2003). Sexual modes questionnaire: measure to assess the interaction among cognitions, emotions, and sexual response. J Sex Res.

[50] Vlaescu G, Alasjö A, Miloff A, Carlbring P, Andersson G (2016). Features and functionality of the Iterapi platform for internet-based psychological treatment. Internet Interv.

[51] Kirana PS, Tripodi F, Reisman Y, Porst H (2013). The EFS and ESSM Syllabus of Clinical Sexology.

[52] Barlow D, Farchione T, Sauer-Zavala S, Latin H, Ellard K, Bullis J (2018). Unified Protocol for Transdiagnostic Treatment of Emotional Disorders: Therapist Guide.

[53] Berking M, Schwarz J (2014). Affect Regulation Training: Handbook of Emotion Regulation.

[54] Berking M, Whitley R (2014). Affect Regulation Training: A Practitioners Manual.

[55] Fritz CO, Morris PE, Richler JJ (2012). Effect size estimates: current use, calculations, and interpretation. J Exp Psychol Gen.

[56] Andrews G, Basu A, Cuijpers P, Craske M, McEvoy P, English C, Newby J (2018). Computer therapy for the anxiety and depression disorders is effective, acceptable and practical health care: An updated meta-analysis. J Anxiety Disord.

[57] Andersson G, Carlbring P, Titov N, Lindefors N (2019). Internet interventions for adults with anxiety and mood disorders: a narrative umbrella review of recent meta-analyses. Can J Psychiatry.

[58] Díaz-García A, González-Robles A, García-Palacios A, Fernández-Álvarez J, Castilla D, Bretón JM, Baños RM, Quero S, Botella C (2021). Negative and positive affect regulation in a transdiagnostic internet-based protocol for emotional disorders: randomized controlled trial. J Med Internet Res.

[59] Meston CM, Buss DM (2007). Why humans have sex. Arch Sex Behav.

[60] Brotto LA, Bitzer J, Laan E, Leiblum S, Luria M (2010). Women's sexual desire and arousal disorders. J Sex Med.

[61] Beck A (1996). Beyond belief: A theory of modes, personality, and psychopathology. Frontiers of Cognitive Therapy.

[62] Nobre PJ, Pinto-Gouveia J (2008). Cognitions, emotions, and sexual response: analysis of the relationship among automatic thoughts, emotional responses, and sexual arousal. Arch Sex Behav.

[63] McCabe MP, Price E (2009). Attrition from an internet-based psychological intervention for erectile dysfunction: who is likely to drop out?. J Sex Marital Ther.

[64] van DS, Sluijs E, van DL, de RD, Heerdink R, Bensing J (2007). Patient adherence to medical treatment: a review of reviews. BMC Health Serv Res.

[65] Donkin L, Glozier N (2012). Motivators and motivations to persist with online psychological interventions: a qualitative study of treatment completers. J Med Internet Res.

[66] Covid-19: casos sobem para 21,6 milhões e mortes, para 602 mil. Agencia Brasil.

[67] Otufowora A, Liu Y, Young H, Egan KL, Varma DS, Striley CW, Cottler LB (2021). Sex differences in willingness to participate in research based on study risk level among a community sample of African Americans in North Central Florida. J Immigr Minor Health.

