# Supplementary material for: Web-Based Emotion Regulation Training for Sexual Health: Randomized Controlled Trial
Source: JMIR Form Res. 2024 Apr 3;8:e50850. doi: 10.2196/50850 (PMC11024748; doi:10.2196/50850)
Supplement: Multimedia Appendix 1 [file formative_v8i1e50850_app1.docx]

Multimedia Appendix 1. Between group comparisons at baseline, end of intervention and 3- month follow-up

Baseline End of intervention 3-month follow-up

|  | Mean (SD) | Mean (SD) | Mean (SD) | Mean (SD) |  | Mean (SD) | Mean (SD) |  |
| --- | --- | --- | --- | --- | --- | --- | --- | --- |
|  | intervention | control p | η^2^ Intervention | control | p | η^2^ intervention | control p | η^2^ |
| FSFI - Desire | 2.90 (1.13) | 2.44 (1.36) 0.429 | 0.76 2.31 (1.64) | 2.91 (1.33) | 1 | 0 2.63(1.48) | 2.91 (1.33) NA | NA |
| FSFI - Arousal | 2.74 (1.38) | 2.83 (1.48) 0.792 | 0.16 2.74 (2.12) | 3.21 (1.480 | NA | NA 2.56(2.02) | 3.21 (1.48) NA | NA |
| FSFI - Lubrification | 2.91 (1.64) | 2.85 (1.64) 0.629 | 0.41 3.56 (2.55) | 3.33 (1.84) | 0.11 | 1.5 2.68(2.25) | 3.33 (1.84) 0.667 | 0.77 |
| FSFI - Orgasm | 2.88 (1.75) | 2.72 (1.66) 0.962 | 0.05 3.09 (2.54) | 2.50 (1.47) | **0.04** | 2,58 2.38(1.91) | 2.50 (1.47) NA | NA |
| FSFI - Satisfaction | 2.87 (1.37) | 2.88 (1.48) 0.711 | 0.33 2.80 (2.15) | 2.84 (1.53) | 0.7 | 0.55 2.64(1.71) | 2.84 (1.52) NA | NA |
| FSFI - Pain | 3.48 (2.20) | 4.07 (2.11) 0.224 | 0.47 2.51 (2.82) | 4.10 (2.02) | 1 | 0 3.14(2.54) | 4.10 (2.02) 0.8 | 0.22 |
| FSFI- Sum | 17.78 (7.29) | 17.79 (7.68) 0.571 | 0.43 17.01 (12.51) | 18.89 (8.09) | 0.38 | 0.79 15.93(10.31) | 18.89 (8.09) NA | NA |
|  |  | 98.60 |  | 101.33 |  |  |  |  |
| DERS - Sum | 95.07 (21.79) | (24.30) 0.403 | 0.22 77.71 (23.70) | (22.05) | 0.42 | 0.32 99.65 (24.36) | 101.33 (22.05) 0.479 | 0.36 |
| DERS - Non  Acceptance | 15.50 (6.73) | 16.20 (7.41) 0.695 | 0.1 11.14 (4.95) | 17.29 (7.25) | 0.87 | 0.07 15.73(6.45) | 17.29 (7.25) 0.616 | 0.24 |
| DERS - Goals | 15.97 (5.12) | 15.83 (4.82) 0.662 | 0.11 12.71 (6.13) | 17.43 (4.30) | 0.21 | 0.5 16.92(4.65) | 17.43 (4.30) 0.616 | 0.26 |
| DERS - Impulse | 13.50 (4.98) | 13.70 (5.03) 0.982 | 0.01 12.00 (4.12) | 14.62 (4.45) | 0.54 | 0.24 14.08(5.39) | 14.62 (4.45) 0.689 | 0.21 |
| DERS - Awareness | 14.43 (4.49) | 14.97 (4.83) 0.486 | 0.18 11.71 (5.91) | 14.71 (4.05) | 0.6 | 0.21 15.65(5.06) | 14.71 (4.05) 0.216 | 0.59 |
| DERS - Strategies | 22.60 (7.92) | 23.93 (7.79) 0.407 | 0.25 19.57 (7.81) | 24.29 (7.44) | 0.63 | 0.19 23.96(8.12) | 24.29 (7.44) 0.842 | 0.12 |
| DERS - Clarity | 13.07 (4.32) | 13.97 (5.53) 0.635 | 0.12 10.57 (4.89) | 13.00 (4.17) | 0.32 | 0.38 13.31(3.79) | 13.00 (4.17) 0.479 | 0.36 |
| EPA - Abuse | 21.47 (7.98) | 15.40 (5.40) **0.004** | 0.81 13.29 (10.08) | 16.33 (4.80) | 0.34 | 0.39 14.65(7.10) | 16.33 (4.80) 0.216 | 0.59 |
| EPA - PassivityControl | 7.93 (3.75) | 6.83 (4.40) 0.153 | 0.37 6.00 (5.26) | 7.71 (4.30) | 0.74 | 0.13 7.65(4.77) | 7.71 (4.30) 0.795 | 0.17 |
| EPA - Negative Self- Image | 14.33 (4.62) | 13.30 (5.26) 0.479 | 0.18 10.29 (8.61) | 14.67 (5.05) | 0.66 | 0.19 13.54(6.06) | 14.67 (5.05) 0.689 | 0.19 |
| EPA - Failure | 8.43 (2.84) | 8.10 (3.27) 0.994 | 0 4.43 (3.50) | 9.33 (2.61) | **0.01** | 1.08 7.46(3.41) | 9.33 (2.61) 0.842 | 0.09 |
| EPA - Lack Partner Attention | 13.20 (4.80) | 10.27 (4.26) **0.025** | 0.61 8.43 (6.70) | 11.76 (4.58) | 0.15 | 0.6 10.69(5.15) | 11.76 (4.59) 0.765 | 0.17 |
| EPA - EroticThoughts | 14.00 (4.31) | 13.73 (4.51) 0.976 | 0.01 10.57 (7.76) | 14.00 (4.00) | 0.9 | 0.05 12.85(5.39) | 14.00 (4.00) 0.921 | 0.05 |
| SSSS - |  | 38.97 |  |  |  |  |  |  |
| Direct/outspoken | 40.80 (9.72) | (11.68) 0.867 | 0.04 29.71 (21.44) | 41.14 (8.28) | 0.18 | 0.59 0 | 41.14 (8.28) 0.546 | 0.31 |
| SSSS - loving/warm | 30.03 (4.49) | 28.47 (8.11) 0.885 | 0.38 22.71 (15.78) | 30.14 (4.14) | 0.14 | 0.62 0 | 30.14 (4.14) 0.179 | 0.65 |
| SSSS -  reserved/conservative | 20.13 (6.05) | 17.57 (4.73) 0.07 | 0.48 13.14 (10.17) | 18.71 (4.51) | 0.55 | 0.25 0 | 18.71 (4.50) 1 | 0 |
| QS - Sexual desire and | 7.27 (3.10) | 7.37 (3.71) 0.636 | 0.12 5.57 (4.61) | 7.19 (4.12) | 0.12 | 0.64 | 7.19 (4.12) 1 | 0 |

| interest |  | | | | | | | | | | |
| --- | --- | --- | --- | --- | --- | --- | --- | --- | --- | --- | --- |
| QS - Foreplay | 3.30 (1.530 | 3.43 (1.47) | 0.596 | 0.13 | 2.14 (1.67) | 3.43 (1.63) | 0.94 | 0.03 | 3.43 (1.630 | 0.305 | 0.49 |
| QS - Arousal and partner connection | 5.90 (2.33) | 5.57 (2.30) | 0.836 | 0.05 | 5.29 (3.77) | 6.14 (2.65) | 0.08 | 0.75 | 6.14 (2.65) | 0.921 | 0.05 |
| QS - Comfort | 6.07 (2.82) | 6.27 (2.77) | 0.619 | 0.13 | 4.71 (4.50) | 6.81 (2.13) | 0.45 | 0.31 | 6.81 (2.14) | 0.146 | 0.73 |
| QS - Orgasm | 4.57 (2.25) | 4.67 (2.79) | 0.714 | 0.09 | 3.29 (2.87) | 4.81 (2.50) | 0.52 | 0.27 | 4.81 (2.50) | 0.689 | 0.19 |
|  |  | 54.60 |  |  |  | 56.76 |  |  |  |  |  |
| QS - Sum | 54.20 (17.37) | (20.07) | 0.4 | 0.22 | 42.00 (30.68) | (21.16) | 0.14 | 0.61 | 56.76 (21.16) | 0.358 | 0.44 |
| PHQ-9Sum | 12.60 (5.75) | 12.67 (6.13) | 0.773 | 0.07 | 11.39 (6.83) | 13.05 (6.55) | 0.12 | 0.64 | 13.05 (6.55) | 0.416 | 0.41 |
| PHQ-9Suicid |  |  | 0.574 | 0.1 |  |  | 0.45 | 0.31 |  | 0.546 | 0.29 |
| PHQ-9Q10 |  |  | 0.785 | 0.06 |  |  | 0.86 | 0.08 |  | 0.842 | 0.09 |
| GAD7Sum | 10.60 (5.37) | 9.33 (4.87) | 0.321 | 0.26 |  |  | 0.89 | 0.07 |  | 0.616 | 0.26 |
