# Supplementary material for: Web-Based Emotion Regulation Training for Sexual Health: Randomized Controlled Trial
Source: JMIR Form Res. 2024 Apr 3;8:e50850. doi: 10.2196/50850 (PMC11024748; doi:10.2196/50850)
Supplement: Multimedia Appendix 2 [file formative_v8i1e50850_app2.pdf]

CONSORT-EHEALTH Checklist V1.6.2 Report	Manuscript Number	50850
**(based on CONSORT-EHEALTH V1.6), available at [http://tinyurl.com/consort-ehealth-v1-6].**		
**Date completed**		
3/30/2024 13:30:04		
**by**		
Vinicius Jobim Fischer		
Web-Based Emotion Regulation Training for Sexual Health: Randomized Controlled Trial		
TITLE		
**1a-i) Identify the mode of delivery in the title**		
"Web-Based Emotion Regulation Training for Sexual Health:"		
**1a-ii) Non-web-based components or important co-interventions in title**		
**1a-iii) Primary condition or target group in the title**		
In order to avoid an extremely long title, we have opted by using the broader term sexual health.		
ABSTRACT		
**1b-i) Key features/functionalities/components of the intervention and comparator in the METHODS section of the ABSTRACT**		
"participants who were randomized to either an Internet-based emotion regulation training for sexual function or to a waitlist control group. Treatment consisted of an 8-week Internet-based emotion regulation training for sexual function. Participants were assessed at baseline, post-intervention, and at 3-months follow-up."		
**1b-ii) Level of human involvement in the METHODS section of the ABSTRACT**		
**1b-iii) Open vs. closed, web-based (self-assessment) vs. face-to-face assessments in the METHODS section of the ABSTRACT**		
**1b-iv) RESULTS section in abstract must contain use data**		
**1b-v) CONCLUSIONS/DISCUSSION in abstract for negative trials**		
INTRODUCTION		
**2a-i) Problem and the type of system/solution**		
"To investigate the effects of an Internet-based emotion regulation training for sexual function in men and women."		
**2a-ii) Scientific background, rationale: What is known about the (type of) system**		
"Effective ER skills are important for sexual function as they impact emotional awareness and expression during sexual activity, and, therefore, satisfaction and distress. Emotion regulation interventions may offer a promising approach to improve sexual health. Internet-based emotion regulation may be a therapeutic strategy for men and women with sexual health concerns. Nevertheless, there is a scarcity of intervention trials investigating their effects in this context, much less using the Internet."		
**Does your paper address CONSORT subitem 2b?**		
"To investigate the effects of an Internet-based emotion regulation training for sexual function in men and women."		
METHODS		
**3a) CONSORT: Description of trial design (such as parallel, factorial) including allocation ratio**		
The final sample included 60 participants who were randomized to either an Internet-based emotion regulation training for sexual function or to a waitlist control group		
**3b) CONSORT: Important changes to methods after trial commencement (such as eligibility criteria), with reasons**		

No changes in eligibility were done		
**3b-i) Bug fixes, Downtimes, Content Changes**		
**4a) CONSORT: Eligibility criteria for participants**		
"Participants were recruited based on their self-reported sexual problems, which for<br>men was defined by a score of < 25 on the International Index Erectile Function (IIEF) and for women by a score of < 26.55 on the Female Sexual Function Index (FSFI)."		
**4a-i) Computer / Internet literacy**		
**4a-ii) Open vs. closed, web-based vs. face-to-face assessments:**		
"participants who were randomized to either an Internet-based emotion regulation training for sexual function or to a waitlist control group."		
**4a-iii) Information giving during recruitment**		
**4b) CONSORT: Settings and locations where the data were collected**		
"...modules delivered via the Internet using a secure Internet-based contact handling system (e.g., similar to Internet banking)."		
**4b-i) Report if outcomes were (self-)assessed through online questionnaires**		
"The International Index of Erectile Function (IIEF)35, is 15-item, self-administered questionnaire for assessing sexual functioning in men" ; "The Female Sexual Function Index (FSFI)37 is a 19-item self-administered questionnaire for the assessment of sexual functioning in women in domains of sexual functioning (e.g., sexual arousal, orgasm, satisfaction, pain)."		
**4b-ii) Report how institutional affiliations are displayed**		
**5) CONSORT: Describe the interventions for each group with sufficient details to allow replication, including how and when they were actually administered**		
**5-i) Mention names, credential, affiliations of the developers, sponsors, and owners**		
**5-ii) Describe the history/development process**		
**5-iii) Revisions and updating**		
**5-iv) Quality assurance methods**		
**5-v) Ensure replicability by publishing the source code, and/or providing screenshots/screen-capture video, and/or providing flowcharts of the algorithms used**		
**5-vi) Digital preservation**		
**5-vii) Access**		
"No compensation was offered to participants." ; "via the Internet using a secure Internet-based contact handling system (e.g., similar to Internet banking)"		
**5-viii) Mode of delivery, features/functionalities/components of the intervention and comparator, and the theoretical framework**		
"Each module consisted of a video presentation, texts, and an exercise (homework) to complete during the week. Access to the intervention modules was granted weekly on the same day and participants were expected to report homework assignments to the therapist until the day prior to the next module. Participant could contact the therapist via the Internet-based platform and expect a reply within 24 hours. No face-to-face or telephone contact with the research team was allowed during the intervention (except for technical problems). Table 1 summarizes the modules contents.<br>Table 1: Summary of intervention modules."		
**5-ix) Describe use parameters**		
**5-x) Clarify the level of human involvement**		

**5-xi) Report any prompts/reminders used**		
"Weekly reminders were sent via email."		
**5-xii) Describe any co-interventions (incl. training/support)**		
no co-intervention was offered.		
**6a) CONSORT: Completely defined pre-specified primary and secondary outcome measures, including how and when they were assessed**		
"The primary outcome measures were IIEF- and FSFI-scores, for men and women respectively." ; "Secondary outcome measures encompassed questionnaires about sexual function, mental health (anxiety and depression), ER, sexual self-perception, and thoughts during sexual activity. Sexual function was assessed with the Sexual Quotient (SQ) – both female38 and male versions." ; "Both primary and secondary outcomes were assessed online on the study website at baseline, end of treatment, and at a 3-month follow-up."		
**6a-i) Online questionnaires: describe if they were validated for online use and apply CHERRIES items to describe how the questionnaires were designed/deployed**		
**6a-ii) Describe whether and how "use" (including intensity of use/dosage) was defined/measured/monitored**		
**6a-iii) Describe whether, how, and when qualitative feedback from participants was obtained**		
**6b) CONSORT: Any changes to trial outcomes after the trial commenced, with reasons**		
"...modules delivered via the Internet using a secure Internet-based contact handling system (e.g., similar to Internet banking)."		
**7a) CONSORT: How sample size was determined**		
**7a-i) Describe whether and how expected attrition was taken into account when calculating the sample size**		
**7b) CONSORT: When applicable, explanation of any interim analyses and stopping guidelines**		
"The primary outcome measures were IIEF- and FSFI-scores, for men and women respectively." ; "Secondary outcome measures encompassed questionnaires about sexual function, mental health (anxiety and depression), ER, sexual self-perception, and thoughts during sexual activity. Sexual function was assessed with the Sexual Quotient (SQ) – both female38 and male versions." ; "Both primary and secondary outcomes were assessed online on the study website at baseline, end of treatment, and at a 3-month follow-up."		
**8a) CONSORT: Method used to generate the random allocation sequence**		
"Two separate block-randomization lists were created via a computer-generated (SealedEnvelope) block-randomization procedure (with a block size of four for two groups) with a 1:1 randomization ratio. The computer-generated sequence was generated by an independent researcher who was not involved in the trial. The actual allocation of participants to the TREPS intervention group or to the waitlist control group was conducted by a clinical psychologist who was not involved in the project, and the researchers had no influence on participants allocation."		
**8b) CONSORT: Type of randomisation; details of any restriction (such as blocking and block size)**		
"Two separate block-randomization lists were created via a computer-generated (SealedEnvelope) block-randomization procedure (with a block size of four for two groups) with a 1:1 randomization ratio. The computer-generated sequence was generated by an independent researcher who was not involved in the trial. The actual allocation of participants to the TREPS intervention group or to the waitlist control group was conducted by a clinical psychologist who was not involved in the project, and the researchers had no influence on participants allocation."		
**9) CONSORT: Mechanism used to implement the random allocation sequence (such as sequentially numbered containers), describing any steps taken to conceal the sequence until interventions were assigned**		
"Two separate block-randomization lists were created via a computer-generated (SealedEnvelope) block-randomization procedure (with a block size of four for two groups) with a 1:1 randomization ratio. The computer-generated sequence was generated by an independent researcher who was not involved in the trial. The actual allocation of participants to the TREPS intervention group or to the waitlist control group was conducted by a clinical psychologist who was not involved in the project, and the researchers had no influence on participants allocation."		
**10) CONSORT: Who generated the random allocation sequence, who enrolled participants, and who assigned participants to interventions**		
"The computer-generated sequence was generated by an independent researcher who was not involved in the trial. The actual allocation of participants to the TREPS intervention group or to the waitlist control group was conducted by a clinical psychologist who was not involved in the project, and the researchers had no influence on participants allocation."		
**11a) CONSORT: Blinding - If done, who was blinded after assignment to interventions (for example, participants, care providers, those assessing outcomes) and how**		
**11a-i) Specify who was blinded, and who wasn't**		

none of the assessors were blinded.		
**11a-ii) Discuss e.g., whether participants knew which intervention was the "intervention of interest" and which one was the "comparator"**		
**11b) CONSORT: If relevant, description of the similarity of interventions**		
No. Pilot innovative intervention.		
**12a) CONSORT: Statistical methods used to compare groups for primary and secondary outcomes**		
"The baseline data present normal univariate distribution according to skewness (-2 to 2) and kurtosis (- 7 to 7). Sociodemographic characteristics of the two groups were compared using Chi-square tests. One-way ANOVAs on outcome variables were performed to examine group differences at baseline. Mann-Whitney U-tests were carried out to compare the two groups (intervention and control) at baseline, at the end of intervention (T1) and at follow-up (T2). Given the large amount of missing data (more than 80%) we did not impute missing data or estimate missing data using mixed models analyses55. All participants who received the intervention and completed the three assessment points were compiled and analyzed for within-group efficacy estimation. A within group non- parametric Fisher test was run to assess pre-post changes. Data were considered significant at a p <.05 level.<br>For parametric analysis, effect size was calculated using Cohen's d. For non-parametric analysis, effect sizes were calculated using η2 56. All statistical analyses were conducted with SPSS version 20."		
**12a-i) Imputation techniques to deal with attrition / missing values**		
"Given the large amount of missing data (more than 80%) we did not impute missing data or estimate missing data using mixed models analyses"		
**12b) CONSORT: Methods for additional analyses, such as subgroup analyses and adjusted analyses**		
"All participants who received the intervention and completed the three assessment points were compiled and analyzed for within-group efficacy estimation. A within group non- parametric Fisher test was run to assess pre-post changes. Data were considered significant at a p <.05 level."		
RESULTS		
**13a) CONSORT: For each group, the numbers of participants who were randomly assigned, received intended treatment, and were analysed for the primary outcome**		
"Comparing the intervention group (IG) (n = 12) and the waitlist control group (WCG) (n = 16), statistically significant differences were found " ; "The intervention effect size calculation was conducted combining all participants who completed the intervention and the 3-month follow-up assessment (n = 5). The results indicated large within-group effect sizes for some of the outcome measures related to improvements ".<br>"The table 3 summarizes the number of participants who read and watched the modules."		
**13b) CONSORT: For each group, losses and exclusions after randomisation, together with reasons**		
the flowchart explain the losses.		
**13b-i) Attrition diagram**		
**14a) CONSORT: Dates defining the periods of recruitment and follow-up**		
No info offered about the recruitment period. "Participants were assessed at baseline, post-intervention, and at 3-months follow-up."		
**14a-i) Indicate if critical "secular events" fell into the study period**		
**14b) CONSORT: Why the trial ended or was stopped (early)**		
the trial ended when finished.		
**15) CONSORT: A table showing baseline demographic and clinical characteristics for each group**		
"Table 2: Participant sociodemographic characteristics."		
**15-i) Report demographics associated with digital divide issues**		
"Table 2: Participant sociodemographic characteristics."		
**16a) CONSORT: For each group, number of participants (denominator) included in each analysis and whether the analysis was by original assigned groups**		
**16-i) Report multiple "denominators" and provide definitions**		

"Comparing the intervention group (IG) (n = 12) and the waitlist control group (WCG) (n = 16), statistically" ; "At the end of the intervention (T1) differences were found regarding orgasm capacity and frequency (IG: n = 6, M = 3.09, SD = 2.54; WCG: n = 10, M = 2.50, SD = 1.47; p =.036; η2 = 2.58)"		
**16-ii) Primary analysis should be intent-to-treat**		
**17a) CONSORT: For each primary and secondary outcome, results for each group, and the estimated effect size and its precision (such as 95% confidence interval)**		
"A within group non- parametric Fisher test was run to assess pre-post changes. Data were considered significant at a p <.05 level."		
**17a-i) Presentation of process outcomes such as metrics of use and intensity of use**		
**17b) CONSORT: For binary outcomes, presentation of both absolute and relative effect sizes is recommended**		
"varied between 14 (n = 2) to 76.6% (n = 23)." ; "Comparing the intervention group (IG) (n = 12) and the waitlist control group (WCG) (n = 16), statistically significant differences were found at baseline regarding the subscales about automatic thoughts of abuse (IG M = 21.47, SD = 7.98; WCG M = 15.40, SD = 5.40; p =.004; d = 0.81) and lack of partner attention (IG M = 13.20, SD = 4.80; p =.025; d = 0.61). At the end of the intervention (T1) differences were found regarding orgasm capacity and frequency (IG: n = 6, M = 3.09, SD = 2.54; WCG: n = 10, M = 2.50, SD = 1.47; p =.036; η2 = 2.58) and automatic thoughts of failure (IG M = 4.43, SD = 3.50, WCG M = 9.33, SD = 2.61, p = .014; η2 = 1.08)."		
**18) CONSORT: Results of any other analyses performed, including subgroup analyses and adjusted analyses, distinguishing pre-specified from exploratory**		
"Sociodemographic characteristics of the two groups were compared using Chi-square tests. One-way ANOVAs on outcome variables were performed to examine group differences at baseline. Mann-Whitney U-tests were carried out to compare the two groups (intervention and control) at baseline, at the end of intervention (T1) and at follow-up (T2)." ; "All participants who received the intervention and completed the three assessment points were compiled and analyzed for within-group efficacy estimation. A within group non- parametric Fisher test was run to assess pre-post changes. Data were considered significant at a p <.05 level.For parametric analysis, effect size was calculated using Cohen's d. For non-parametric analysis, effect sizes were calculated using η2 56."		
**18-i) Subgroup analysis of comparing only users**		
**19) CONSORT: All important harms or unintended effects in each group**		
no harms or unintended effects applicable.		
**19-i) Include privacy breaches, technical problems**		
**19-ii) Include qualitative feedback from participants or observations from staff/researchers**		
DISCUSSION		
**20) CONSORT: Trial limitations, addressing sources of potential bias, imprecision, multiplicity of analyses**		
**20-i) Typical limitations in ehealth trials**		
"First and foremost, the high dropout rate precludes any significant conclusions to be drawn from the results. Significant attrition should be addressed as a risk factor in future similar Internet-based ER training for sexual function. In addition, and in contrast to the potential advantages of online interventions (e.g., anonymity, ease of access etc.) the high dropout rates in programs of this kind may indicate a serious limitation to the suitability of Internet-trainings concerning sexual health. Second, most participants were highly educated, which affects the generalizability of the results and their scalability. Third, since the inclusion criteria were based on self-report measures, response bias and possible failures to detect interfering medical conditions not known by the participants may have occurred. Fourth, we did not assess participants' expectations with regards to the effects of the training, so it remains unclear as to whether these impacted the results."		
**21) CONSORT: Generalisability (external validity, applicability) of the trial findings**		
**21-i) Generalizability to other populations**		
**21-ii) Discuss if there were elements in the RCT that would be different in a routine application setting**		
**22) CONSORT: Interpretation consistent with results, balancing benefits and harms, and considering other relevant evidence**		
**22-i) Restate study questions and summarize the answers suggested by the data, starting with primary outcomes and process outcomes (use)**		

The present study aimed to investigate the effects of an Internet-based ER training for sexual function. The trial suffered from a substantial dropout rate, but the preliminary per protocol results suggest that the TREPS protocol provided some improvements in relation to sexual function, however mainly for mental health and ER abilities. Although no significant effects were seen in the main outcome measures after the 3-month follow-up in the controlled between group analyses, large and moderate within-group effect sizes were found for a range of components in the within group analyses.		
**22-ii) Highlight unanswered new questions, suggest future research**		
Other information		
**23) CONSORT: Registration number and name of trial registry**		
ClinicalTrials.gov (NCT04792177).		
**24) CONSORT: Where the full trial protocol can be accessed, if available**		
https://link.springer.com/article/10.1186/s13063-021-05586-x		
**25) CONSORT: Sources of funding and other support (such as supply of drugs), role of funders**		
The SHER 2 study has received financial support from Luxembourg National Research Fund (FNR), under the grant number: 11606105 The funder of the study had no role in the study design and preparation and will have no role in the data collection, analysis, and interpretation or writing of the publications.		
**X26-i) Comment on ethics committee approval**		
**x26-ii) Outline informed consent procedures**		
**X26-iii) Safety and security procedures**		
**X27-i) State the relation of the study team towards the system being evaluated**		
